# Post-transcriptional regulation of light-stress responses and predictive modeling in vegetable *Solanaceae* Crops

**DOI:** 10.3389/fpls.2026.1880044

**Published:** 2026-07-07

**Authors:** Lidiia Samarina, Farida Kozhakhmetova, Arysgul Turbekova, Serik Jantassov, Yerzhan Syrgaliyev, Amangeldy Taukenov, Valery Stolyarov, Halil Demir

**Affiliations:** 1 Saken Seifullin Kazakh Agrotechnical University, Astana, Kazakhstan; 2 Kazakh Research Institute of Fruit and Vegetable Growing LLP, Almaty, Kazakhstan; 3 Led System Media, Astana, Kazakhstan; 4 Department of Horticulture, Faculty of Agriculture, University of Akdeniz, Antalya, Türkiye

**Keywords:** abiotic stress, alternative splicing, light signaling, machine learning, small RNAs, Solanaceae, translational control

## Abstract

Vegetable *Solanaceae* crops—tomato, pepper, eggplant, and potato—are increasingly cultivated in controlled environments where light is managed both as an energy source and as a developmental and stress-regulatory signal. Programmable spectra, photoperiods, and intensities interact with heat, drought, salinity, chilling, and nutrient limitation, generating complex physiological responses that cannot be explained by transcriptional regulation alone. This review highlights post-transcriptional RNA regulation as a key interface linking light perception with stress adaptation in vegetable *Solanaceae*. We focus on four regulatory layers—alternative splicing, RNA stability and decay, small RNAs pathways, and translational control—that determine which transcripts are processed, stabilized, degraded, or translated under specific environmental histories. Evidence from tomato, pepper, and potato indicates that RNA-level regulation contributes to stress responses, developmental flexibility, and genotype-specific acclimation. However, direct mechanistic links between defined photoreceptor pathways and specific post-transcriptional processes remain limited in *Solanaceae*; therefore, mechanisms established in *Arabidopsis* and other model plants are treated here as testable hypotheses rather than confirmed crop mechanisms. We further discuss how machine learning can integrate multi-omics and environmental datasets to identify predictive regulatory modules connecting light regimes with stress resilience and crop performance. Progress in this field will depend on experiments that combine precise light and microclimate monitoring with isoform-resolved transcriptomics, small RNAs/degradome analyses, RNA stability measurements, and translatome profiling. Such integration can transform controlled-environment *Solanaceae* research from descriptive stress omics to predictive, mechanism-based crop management.

## 1 Introduction

Vegetable crops of the *Solanaceae* family, including tomato (*Solanum lycopersicum*), sweet pepper (*Capsicum annuum*), eggplant (*Solanum melongena*) and potato (*Solanum tuberosum*), are central to global horticulture and are increasingly cultivated in protected and controlled-environment systems. The importance of *Solanaceae* crops is reflected in the U.S. Census of Horticultural Specialties, where food crops grown under protection generated USD 703.5 million in sales in 2019; tomatoes alone accounted for USD 345.0 million, 2,205 operations and 52,576 thousand ft² protection, while peppers were also reported among protected food crops (USDA-NASS, 2019). In controlled-environment agriculture, artificial lighting systems allow growers to manipulate spectral quality, intensity, photoperiod, light direction and timing with increasing precision. However, light regimes that promote growth under one condition may become stressful under another, especially when plants experience excessive irradiance, spectral imbalance, prolonged photoperiods, high blue-light fractions, far-red enrichment or supplemental UV-B. In fruiting *Solanaceae* crops, these light conditions influence canopy structure, source–sink balance, flowering, fruit development, ripening, pigment accumulation and stress acclimation (Heuvelink et al., 2025). Changes in spectral composition alter not only photoreceptor activation states but also photosynthetic carbon gain, temperature, transpiration, redox balance, and hormone signaling. These downstream shifts influence how plants respond to stress factors such as nutrient limitation, salinity, heat, or chilling.

**Figure 1:**
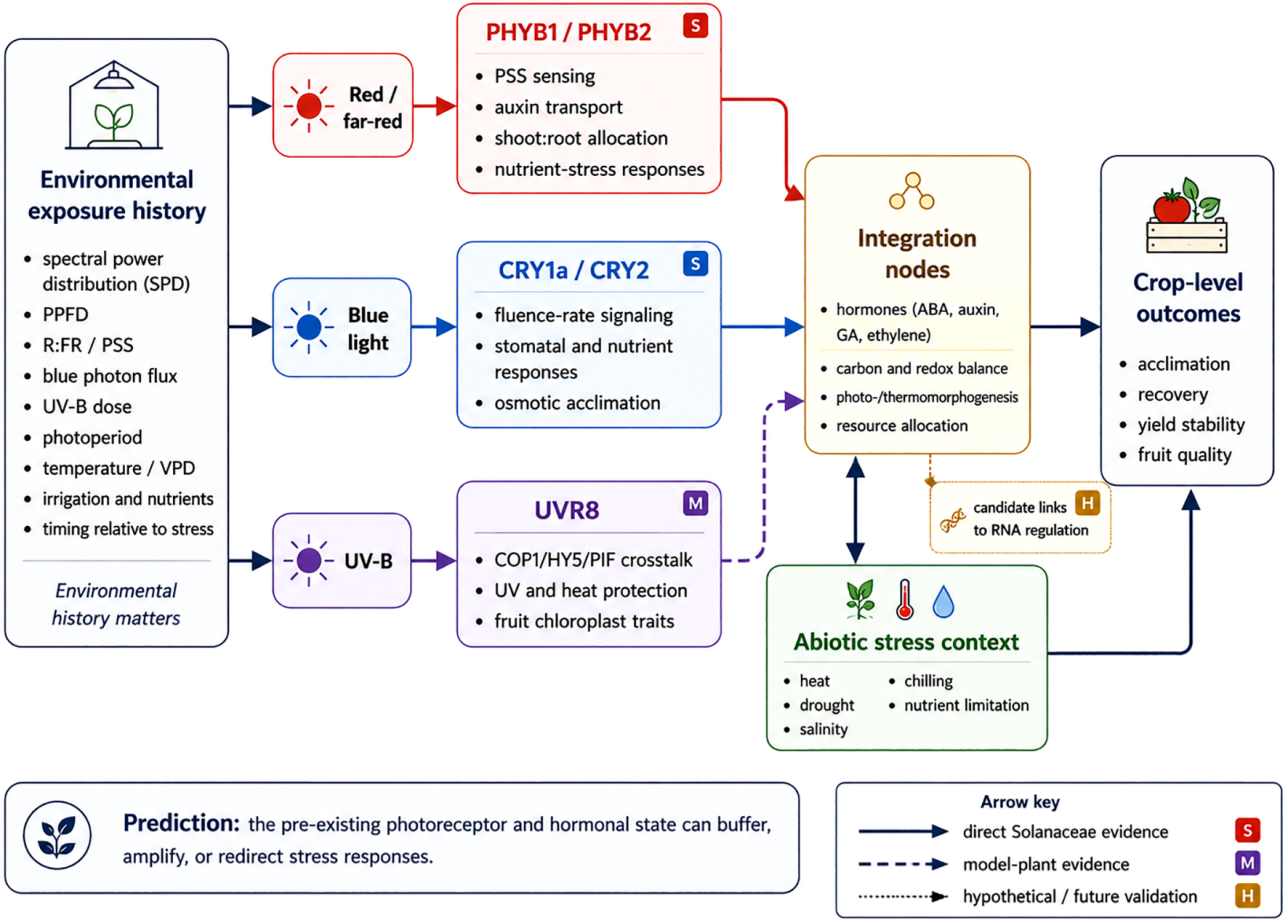
Evidence-resolved framework linking controlled-environment light perception with *Solanaceae* stress physiology. Programmable red/far-red, blue and UV-B radiation establish photoreceptor activation states through PHYB1/PHYB2, CRY1a/CRY2 and UVR8, respectively. These signals converge through hormonal, carbon, redox and developmental integration nodes to influence stress acclimation, recovery, yield stability and fruit quality. Solid arrows indicate relationships supported by direct *Solanaceae* evidence, including phytochrome-dependent far-red responses, CRY1a-associated osmotic and nutrient-stress responses, and UVR8-dependent UV-B acclimation or fruit traits. Dashed arrows indicate mechanisms mainly supported by model-plant evidence, such as UV-B/COP1/HY5/PIF-related crosstalk, while dotted arrows indicate proposed *Solanaceae*-relevant links requiring direct validation. S, M and H denote direct *Solanaceae* evidence, model-plant evidence and hypothetical/future validation, respectively.

Plants perceive light through specialized photoreceptor families. Phytochromes sense red and far-red radiation, cryptochromes respond mainly to blue light, and UV RESISTANCE LOCUS 8 (UVR8) perceives UV-B radiation. These receptors coordinate photomorphogenesis, shade-avoidance responses, stomatal behavior, chloroplast development, circadian regulation, flowering and protective acclimation. In tomato, far-red radiation modifies shoot–root biomass allocation through PHYB1/PHYB2-dependent signaling and auxin transport, showing that spectral cues can reorganize whole-plant resource distribution in ways directly relevant to productivity (Ji et al., 2023). Phytochromes mediate far-red-dependent biomass allocation and interact with nutrient-stress pathways (Ji et al., 2023; Soares et al., 2021); CRY1a participates in osmotic and nutrient-deficiency responses under specific blue-light intensities (D’Amico-Damião et al., 2022); and UVR8 regulates UV-B tolerance while modulating shade and thermomorphogenic responses (Liu et al., 2020; Hwang et al., 2025). This framework predicts pronounced non-additive effects under combined conditions such as far-red × heat, blue × salinity, UV-B × temperature, or spectrum × nutrient limitation. It also highlights the need for comprehensive reporting of environmental histories rather than broad treatment labels (**Figure 1**). Far-red effects also depend on the balance between photosynthetic energy input and phytochrome-mediated signaling, indicating that spectral responses in greenhouses cannot be interpreted only through classical shade-avoidance models (Shomali et al., 2024).

Most studies on light responses in *Solanaceae* crops have focused on photosynthesis, morphology, hormone signaling and transcriptional regulation. These mechanisms are essential, but they do not fully explain how plants adjust rapidly to changing light environments. Transcript abundance alone often fails to predict physiological output because gene expression is also controlled after transcription. Alternative splicing, RNA stability and decay, small RNAs pathways and translational control determine which transcripts are processed, retained, degraded, stored or translated. These regulatory layers can reshape gene-expression outcomes within short time scales and may be especially important under fluctuating light regimes, where plants must repeatedly shift between growth, acclimation and recovery (Xu et al., 2024a; Cai et al., 2024).

Thus, the specific knowledge gap addressed in this review is that post-transcriptional regulation has not yet been systematically synthesized as a central component of light-stress biology in vegetable *Solanaceae*. Direct mechanistic links between defined photoreceptor pathways and RNA-level regulation remain limited in tomato, pepper, eggplant and potato. At the same time, RNA-level processes contribute to stress responsiveness, developmental plasticity and genotype-specific acclimation in these crops. In parallel, model-plant studies have revealed direct connections between photoreceptors and RNA regulation, including phytochrome-associated splicing factors, CRY2-dependent alternative splicing, COP1-associated RNA processing, light-regulated RNA stability and light-dependent translational control (Kathare and Huq, 2021; Kathare et al., 2022; Zhao et al., 2022; Li et al., 2022). The challenge is how these mechanisms operate under the programmable light environments used for vegetable *Solanaceae* production.

Thus, in this review, we synthesize current knowledge on how light perception may interact with post-transcriptional RNA regulation in vegetable *Solanaceae* crops. The need for this review is reinforced by three recent developments. First, LED-based cultivation now allows specific light regimes to be designed and reproduced with high precision, making it possible to test light-quality effects at the molecular level. Second, isoform-resolved transcriptomics, small RNAs/degradome analysis, RNA-stability assays and ribosome profiling now provide tools to measure RNA regulation beyond total transcript abundance. Third, machine-learning approaches can integrate environmental metadata, multi-omics datasets and crop phenotypes to identify predictive regulatory modules connecting light regimes with plant performance (Libbrecht and Noble, 2015; Mahood et al., 2020; Panahi et al., 2025). Together, these advances create an opportunity to move controlled-environment *Solanaceae* research from descriptive light-response studies toward predictive, mechanism-based crop management.

## 2 Alternative splicing as a light-responsive regulatory layer in vegetable *Solanaceae crops*

The concept of light-regulated AS is supported by several complementary lines of evidence. Early genome-wide work in the moss *Physcomitrium patens* showed that light induces widespread AS changes, particularly intron-retention events in transcripts associated with photosynthesis and translation (Wu et al., 2014). In *Arabidopsis*, phytochrome signaling has been directly connected to AS regulation. Shikata et al. (2014) demonstrated that phytochromes control AS profiles at the genomic level during light responses. Subsequent studies identified splicing-related proteins that interact with phytochrome signaling. The SFPS–RRC1 complex was shown to coordinate pre-mRNA splicing of genes involved in light signaling and circadian regulation, thereby promoting photomorphogenesis (Xin et al., 2019). Later, the SWAP1–SFPS–RRC1 splicing-factor complex was shown to interact with photoactivated phyB and modulate AS to promote photomorphogenesis (Kathare et al., 2022).

Blue-light signaling also intersects with AS regulation. In *Arabidopsis*, CRY2 interacts with CRY2 INTERACTING SPLICING FACTOR 1 (CIS1) in a blue-light-dependent manner and regulates thermosensory flowering through AS of *FLOWERING LOCUS M* (*FLM*) (Zhao et al., 2022). COP1-centered signaling provides another route through which light may regulate AS. COP1 is a central repressor of photomorphogenesis in darkness and integrates signals from multiple photoreceptor pathways. Li et al. (2022) showed that COP1 and the RNA helicase UAP56 coordinately regulate AS to repress photomorphogenesis in Arabidopsis. More recently, light was shown to regulate nuclear detainment of intron-retained transcripts through a COP1–spliceosome pathway, affecting transcripts of light-signaling genes such as *PIF4*, *RVE1*, and *ABA3* (Zhou et al., 2024a). Intron-retained transcripts may be retained in the nucleus, preventing premature translation and allowing light conditions to control whether specific mRNA isoforms become functional. Light can also regulate AS indirectly through photosynthesis-derived signals. Godoy Herz et al. (2019) showed that light influences AS through changes in RNA polymerase II elongation associated with chloroplast retrograde signaling. Riegler et al. (2021) further demonstrated that light-regulated AS in roots can be mediated through photosynthesized sugars and the TARGET OF RAPAMYCIN (TOR) kinase pathway.

Among vegetable *Solanaceae* crops, tomato is currently the strongest platform for investigating AS because of its high-quality genome resources, extensive transcriptome datasets, available photoreceptor mutants, and direct relevance to greenhouse production. Genome-wide integration of tomato mRNA, expressed sequence tags, and RNA-seq datasets identified 369,911 AS events from 34,419 genomic loci involving 161,913 transcripts (Clark et al., 2019). In that analysis, 23,233 of 35,768 annotated protein-coding gene models generated AS isoforms, corresponding to an estimated AS rate of approximately 65% in tomato. Intron retention was the most frequent basic AS event, followed by alternative acceptor-site and donor-site events, while exon skipping was less frequent (Clark et al., 2019). Wang et al. (2017) compared tomato seedlings grown in a phytotron and a plastic greenhouse and found that different growth environments altered both gene expression and AS patterns. Because these environments differed in light conditions, temperature, and other microclimatic variables, the study cannot be interpreted as evidence for direct spectral regulation of AS. However, it is highly relevant to controlled-environment research because it shows that tomato AS is environmentally plastic and genotype dependent. Importantly, the study also suggested that AS–NMD coupling may contribute to gene-expression regulation under fluctuating environments (Wang et al., 2017).

The best-supported candidate route for light-regulated AS in vegetable *Solanaceae* is red/far-red control through phytochrome-associated splicing factors. In Arabidopsis, phyB interacts with splicing regulators and affects AS during photomorphogenesis (Shikata et al., 2014; Xin et al., 2019; Kathare et al., 2022). Tomato is an appropriate crop system in which to test this model because red and far-red light are widely used in greenhouse lighting to manipulate elongation, biomass allocation, flowering, and fruit development. If conserved, phytochrome-regulated AS could provide a mechanism through which far-red supplementation modifies crop architecture and source–sink allocation beyond transcriptional regulation alone. In practical terms, far-red treatments could be tested in tomato PHYB1/PHYB2 mutant or silenced backgrounds using isoform-resolved transcriptomics. Candidate outputs would include ΔPSI values, intron-retention frequency, splice-junction usage, AS–NMD coupling, and full-length isoform ratios. Genes of particular interest would include those encoding transcription factors, auxin transport or signaling components, circadian regulators, flowering regulators, and RNA-processing factors. If red/far-red regimes alter these isoform profiles in wild-type plants but not in phytochrome-deficient backgrounds, this would support direct phytochrome-dependent AS regulation in tomato.

A second candidate route is blue-light regulation through cryptochrome-associated splicing modules. The Arabidopsis CRY2–CIS1–*FLM* mechanism demonstrates that blue light can regulate flowering through AS (Zhao et al., 2022). In tomato and pepper, flowering and fruit set are key determinants of productivity under protected cultivation. Although tomato does not use the same vernalization-dependent flowering system as Arabidopsis, cryptochrome-regulated AS may still affect crop-relevant developmental transitions through analogous regulators of flowering, circadian timing, hormone signaling, or meristem identity. Blue-light fluence rate is routinely adjusted in CEA to control plant compactness, leaf anatomy, stomatal behavior, and pigment accumulation. Therefore, testing whether blue-light fraction or fluence modifies AS profiles in CRY-dependent pathways is a logical priority.

A third candidate route involves COP1-associated spliceosome regulation. COP1 is positioned downstream of multiple photoreceptors, including phytochromes, cryptochromes, and UVR8. Arabidopsis studies show that COP1 can interact with RNA-processing machinery and influence AS, including intron-retained transcript detainment (Li et al., 2022; Zhou et al., 2024a). For *Solanaceae* crops, this suggests that red, blue, and UV-B light could converge on shared RNA-processing outputs through COP1-related pathways. In tomato, where UV-B and blue-light treatments are increasingly used to regulate morphology, antioxidant capacity, and fruit quality, COP1-associated AS should be considered a plausible mechanism requiring direct validation.

A fourth route is photosynthesis- and sugar-dependent AS regulation. The light–sugar–TOR pathway described in Arabidopsis roots (Riegler et al., 2021) provides a particularly relevant framework for fruiting crops. Tomato fruit load, leaf photosynthetic capacity, and sink demand interact strongly with lighting strategies. Therefore, some AS responses to light may be systemic rather than local: illuminated leaves may produce carbohydrate or retrograde signals that alter AS in roots, meristems, flowers, or developing fruits. This possibility is especially important when interpreting AS data from whole seedlings or mixed tissues, because tissue-specific signals may be obscured.

AS outcomes depend on splice-site strength, RNA secondary structure, transcriptional elongation rate, chromatin environment, and the abundance or activity of splicing regulators. In plants, serine/arginine-rich proteins, heterogeneous nuclear ribonucleoproteins, RNA helicases, and other auxiliary splicing factors influence splice-site choice. In tomato, the serine/arginine-rich protein family has been systematically characterized. Rosenkranz et al. (2021) identified 17 canonical SR and two SR-like genes in tomato and showed that several SR genes display differential expression and altered splicing profiles across organs and under high temperature. More recently, a plant-specific clade of SR proteins, including RS2Z35 and RS2Z36, was shown to regulate splicing homeostasis and thermotolerance in tomato, with broad effects on heat-sensitive AS events (Rosenkranz et al., 2024). They also identify tomato splicing regulators that could be tested under defined light spectra. A large-scale analysis of *Capsicum annuum* RNA-seq datasets identified extensive AS variation across tissues and stress-related conditions, generating a useful splice-variant resource for hot pepper (Kim et al., 2024). Although these data are not centered on controlled light treatments, they expand the *Solanaceae* comparative framework and can support future studies testing whether tomato and pepper share conserved light-responsive AS signatures.

## 3 RNA stability, decay, and condensates: post-transcriptional “traffic control” of stress programs under light cues

**Figure 2:**
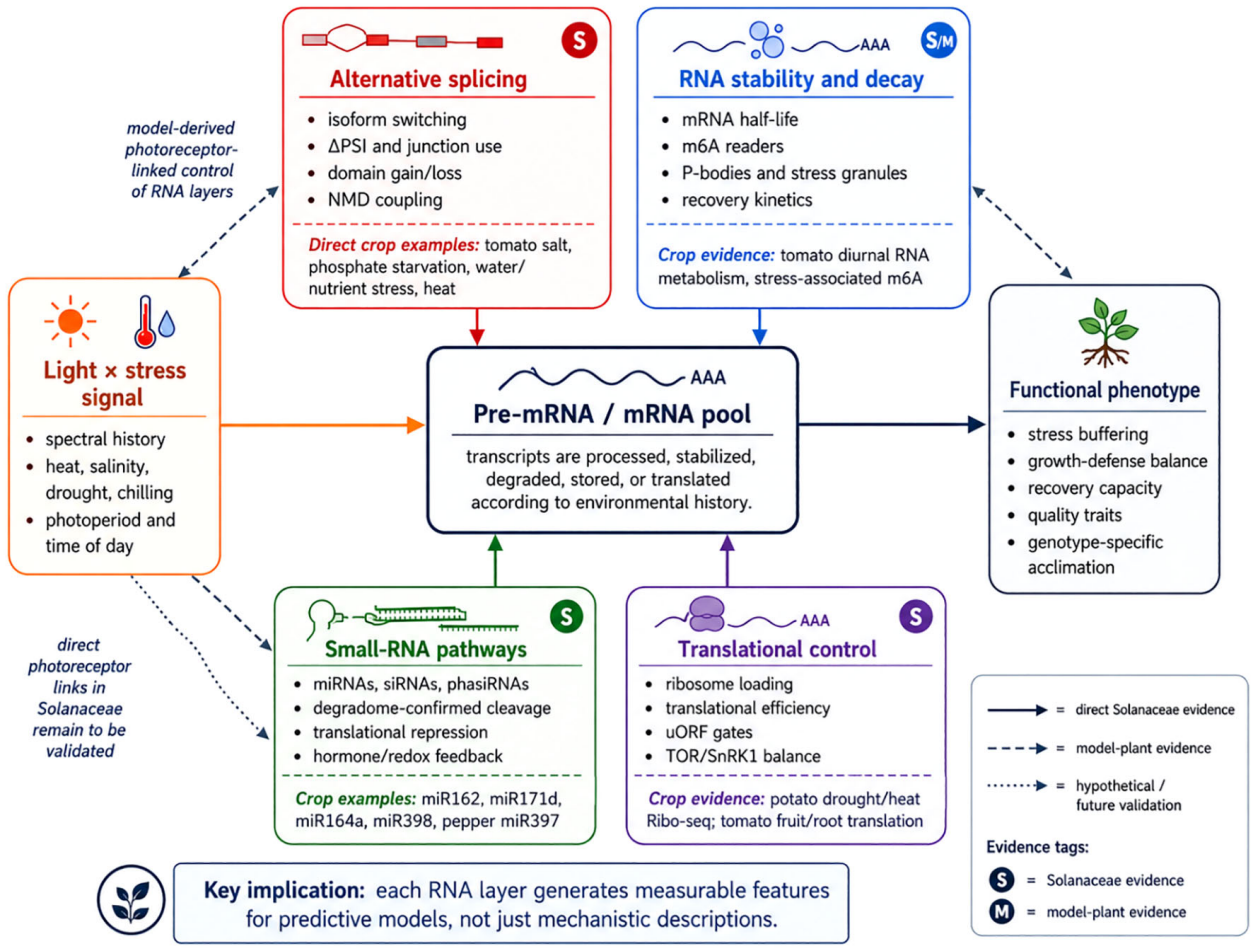
Evidence-resolved model of post-transcriptional RNA-regulatory layers connecting light -stress signals to *Solanaceae* phenotypes. Alternative splicing, RNA stability and decay, small RNAs pathways and translational control influence whether transcripts are processed, stabilized, degraded, stored or translated under specific environmental histories. Solid arrows indicate RNA-regulatory relationships supported by direct *Solanaceae* evidence, including stress-responsive alternative splicing in tomato, stress-associated miRNA modules in tomato and pepper, and translational reprogramming in potato and tomato tissues. Dashed arrows indicate model-derived mechanisms, including photoreceptor-associated splicing regulation, COP1-associated RNA regulation, DCP5/ECT8-mediated RNA decay and phytochrome-linked P-body dynamics. Dotted arrows indicate proposed direct photoreceptor-to-RNA links in *Solanaceae* that require validation using defined spectra, photoreceptor mutants and matched RNA-level assays. S and M indicate *Solanaceae* and model-plant evidence, respectively.

The stability and decay of mRNA therefore act as key control points in gene-expression networks, especially in fluctuating environments typical of greenhouse and vertical-farming systems (**Figure 2**). Direct *Solanaceae* evidence supports a role for RNA metabolism in tomato diurnal transcript dynamics and for stress-associated m^6^A remodeling in tomato fruit, indicating that RNA fate contributes to crop-relevant stress and developmental responses (Zhang et al., 2023; Han et al., 2025). However, the detailed mechanistic links between photoreceptors, RNA half-life, P-body/stress-granule behavior and condensate-mediated RNA fate have been resolved mainly in model systems. *Arabidopsis* studies on DCP5-dependent condensates, ECT8-mediated m^6^A-dependent decay, stress-granule recovery functions and far-red-associated P-body disassembly therefore provide mechanistic models for targeted validation in *Solanaceae* crops (Wang et al., 2024; Cai et al., 2024; Schwenk and Hiltbrunner, 2022).

Studies in tomato have shown that diurnal cycles of transcript levels are strongly influenced by RNA metabolism, establishing that oscillations in mRNA abundance depend as much on degradation rate as on transcriptional activity (Zhang et al., 2023). A revealing example comes from the DCP5 protein, a component of the PB decapping complex. In *Arabidopsis*, DCP5 contains a plant-specific, crowding-sensitive region that senses osmotic stress and undergoes conformational change to promote phase separation into DCP5-rich condensates. These structures sequester mRNAs and regulatory proteins, rewiring the transcriptome and translatome for osmotic adjustment (Wang et al., 2024). Although directly demonstrated in *Arabidopsis*, this model is highly relevant to *Solanaceae*, where greenhouse stress episodes frequently involve transient drought or salinity. Proximity-biotinylation and RNA-capture experiments have revealed that PB-associated RNAs shift according to physiological state; different stress conditions lead to selective enrichment of distinct transcript sets (Wu et al., 2024). PBs therefore function as sorting stations rather than inert storage sites. For *Solanaceae* crops, this implies that tolerance may depend on whether transcripts encoding ion transporters, antioxidant enzymes, or repair proteins are retained for later translation or funneled into decay pathways. Collectively, PB and SG regulation constitutes a molecular “traffic-control” system coordinating transcript use, storage, and clearance during stress cycles.

RNA modifications introduce an additional dimension of post-transcriptional regulation. Among them, N^6^-methyladenosine (m^6^A) plays a central role in defining RNA fate by influencing stability, translation, or localization through interaction with specific reader proteins. Under abiotic stress, m^6^A patterns can be rapidly remodeled, providing a mechanism for plants to reprioritize mRNA fate. In *Arabidopsis*, the reader protein ECT8 acts as a salt-stress sensor that binds methylated mRNAs and promotes their degradation by recruiting the decapping component DCP5 (Cai et al., 2024). In tomato, stress-dependent reshaping of m^6^A landscapes has been documented in fruit tissues exposed to drought, salinity, heat, and cold. m^6^A enrichment correlates with the stability of transcripts involved in cell-cycle control, auxin and ethylene signaling, and carotenoid metabolism, notably lycopene accumulation (Han et al., 2025).

Comprehensive analyses encompassing transcription, decay, and translation during light-stress show that light directly modulates mRNA half-life and translational efficiency (Smith et al., 2024). Hence, photosynthetic or redox perturbations triggered by changes in light intensity provoke global rearrangements of RNA fate. These findings justify investigating whether particular spectra—such as far-red enrichment, high blue fractions, or supplemental UV-B—affect RNA half-life or PB/SG partitioning in a photoreceptor-dependent manner. A particularly intriguing observation is that phytochrome A mediates PB disassembly under far-red light (Schwenk and Hiltbrunner, 2022). Because far-red supplementation is common in greenhouse lighting, this suggests a direct physical mechanism by which specific light spectra could regulate mRNA storage and release. If similar processes operate in *Solanaceae*, far-red light could influence stress recovery not only through morphological signals but also via the controlled mobilization of RNA pools from PBs to translation. Considering that lighting schedules in CEA can be precisely controlled, understanding and manipulating RNA-decay and condensate pathways could open new strategies for real-time stress management and yield stabilization.

## 4 Small RNAs (miRNAs/siRNAs/phasiRNAs) as post-transcriptional integrators of light and stress in vegetable *Solanaceae*

Direct *Solanaceae* evidence supports the functional importance of several stress-responsive miRNA modules, including tomato miR162–DCL1 under low-night-temperature stress, miR171d–GRAS24 in fruit chilling response, miR164a in salinity tolerance, miR398 in thermotolerance and pepper miR397–laccase in anther heat response (Xing et al., 2022; Li et al., 2023; Wan et al., 2023; Li et al., 2025; Lei et al., 2026). Multiple functionally characterized miRNA–target pairs regulate responses relevant to controlled-environment productivity, postharvest life, and quality traits (Xing et al., 2022; Li et al., 2023; Wan et al., 2023; Bansal et al., 2024; Li et al., 2025; Duan et al., 2026). In potato, combined drought and heat stresses trigger cultivar-specific miRNA responses, while low-temperature treatments alter the abundance of known and novel miRNAs in leaves and roots (Gökçe et al., 2021; Yan et al., 2021). In eggplant, small RNAs sequencing has identified conserved and novel miRNAs, and integrated miRNA–mRNA analyses have been used to characterize responses to waterlogging and pathogen stress (Yang et al., 2013; Jiang et al., 2023a). These studies establish small RNAs as experimentally supported regulators of stress physiology in *Solanaceae*.

sRNA activity must be viewed within the physiological background defined by light intensity, spectral balance, and photoperiod. Light quality regulates photosynthetic electron flow, reactive oxygen species (ROS) generation, stomatal behavior, and hormone levels; these factors in turn shape *MIR* transcription, DICER-LIKE 1 (DCL)/ARGONAUTE proteins activity, and target-site accessibility. Because abiotic-stress signaling in *Solanaceae* depends heavily on abscisic acid (ABA), auxin, ethylene, and jasmonate pathways, small RNAs are experimentally supported regulators of stress physiology in *Solanaceae* and plausible candidates for connecting light-regulated redox, hormone and carbohydrate states with post-transcriptional stress responses (Song et al., 2019; Gulyás et al., 2023).

Red-to-blue light transition in tomato has been associated with altered miRNA expression (Dong et al., 2020), while red-light signaling in *Arabidopsis* has been mechanistically linked to *DCL1*, *PIF4* and miRNA biogenesis (Sun et al., 2018). In parallel, high-light and spectral changes can modify plastid and redox signaling, which in turn can affect miRNA regulation (Gulyás et al., 2023). In tomato, the miR162–DCL1 module influences ABA-associated stomatal conductance under low-night-temperature stress (Li et al., 2023).

Expanded identification of MIR172 loci and a novel SEC14-like phosphatidylinositol transfer-protein target in tomato revealed that miR172 regulates stress responses through both cleavage and translational repression (Bansal et al., 2024). Such regulatory flexibility is advantageous under fluctuating environmental conditions, allowing rapid shifts in protein output without eliminating transcript pools entirely. Reproductive tissues are particularly sensitive to heat, and sRNA regulation appears central to maintaining pollen viability and fruit set in pepper. High temperatures compromise anther development through disruptions in cell-wall remodeling, hormone balance, and carbohydrate transport—processes tightly controlled by miRNAs (Song et al., 2019; Lei et al., 2026).

In model plants, small RNAs mediate crosstalk between UV-B and pathogen signaling pathways, showing that miRNAs can coordinate light-responsive and stress-responsive networks within shared regulatory hierarchies (Zhou et al., 2024b). Likewise, light-dependent redox shifts modulate miRNA abundance and activity (Gulyás et al., 2023). These findings justify targeted testing in tomato, pepper, and potato: for instance, assessing whether far-red enrichment alters miRNAs related to auxin transport, whether blue-light fluence modifies osmotic-stress miRNAs, or whether UV-B supplementation regulates antioxidant or fruit-quality-associated miRNAs.

**Figure 3:**
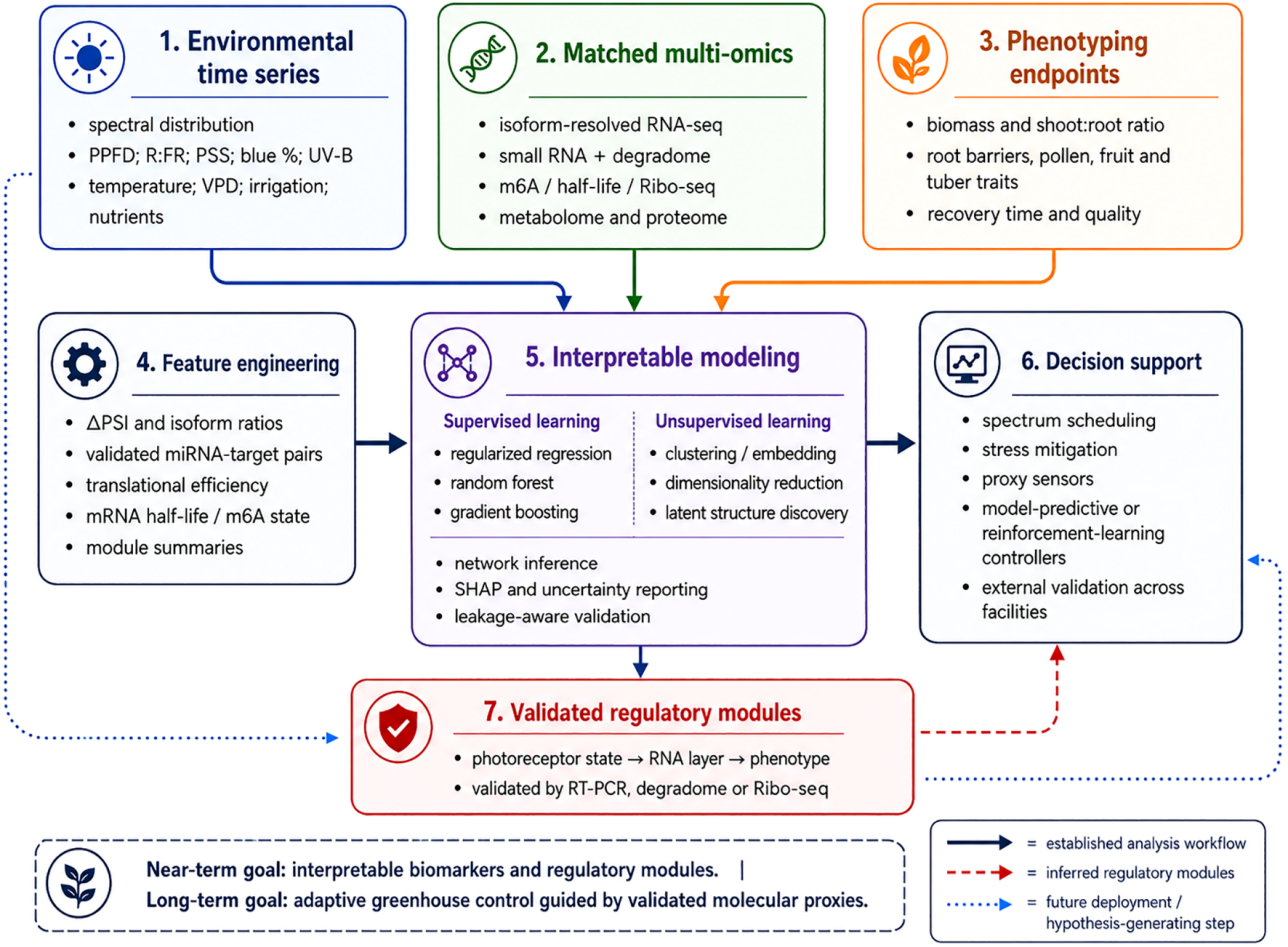
Interpretable machine-learning framework for integrating light regimes, RNA regulation and crop performance in *Solanaceae*. Environmental time series, matched multi-omics and phenotyping endpoints are converted into biologically interpretable features, including isoform ratios, ΔPSI values, validated miRNA–target pairs, RNA-stability indicators, m^6^A state, translational-efficiency values and environmental time-window summaries. Supervised learning can be used for prediction of stress class, stress severity or recovery traits, whereas unsupervised learning can identify hidden structure, treatment gradients and regulatory modules. Solid arrows indicate the established analytical workflow from data acquisition to feature engineering, modeling and validation. Dashed arrows indicate inferred regulatory modules requiring biological confirmation, while dotted arrows indicate future deployment or hypothesis-generating links toward decision support. Model interpretation should include feature-importance analysis, SHAP values, leakage-aware validation, external testing and uncertainty reporting.

Because they often target regulatory hubs rather than terminal effector genes, small RNAs signatures are well suited for integration into machine-learning (ML) models aimed at predicting stress resilience (**Figure 3**). Features such as miRNA abundance, validated miRNA–mRNA pairs, target-cleavage evidence, translational-repression indices, and environmental metadata can be combined to identify predictive modules of tolerance and recovery (Bansal et al., 2024; Song et al., 2019; Lei et al., 2026). Given that miRNA activity can shift with redox and hormone status, both influenced by light, integrating sRNA and environmental dynamics will help define predictive molecular markers for stress tolerance.

Overall, small RNAs pathways represent versatile and rapid-acting regulators that mediate cross-talk between light perception, hormone signaling, oxidative balance, and abiotic stress adaptation in vegetable *Solanaceae*. Their capacity to operate through both transcript cleavage and translation inhibition allows fine-grained control of gene expression under fluctuating light and environmental conditions (Li et al., 2013; Yu et al., 2017). The next challenge lies in demonstrating direct photoreceptor dependence and in leveraging miRNA signatures within integrated multi-omics and predictive frameworks to guide spectrum design and stress-management strategies in controlled-environment agriculture.

## 5 Translational control under fluctuating light and stress in vegetable *Solanaceae*

Light regime, as both an energy source and environmental cue, affects photosynthetic output, redox balance, and carbohydrate status—all of which influence translation through central metabolic regulators, including TARGET OF RAPAMYCIN (TOR) and SNF1-RELATED KINASE 1 (SnRK1) (Baena-González and Hanson, 2017; Wu et al., 2024a). The distinction between transcript abundance and protein synthesis has profound implications for stress biology. Many stress-induced transcripts are translationally inactive, whereas other mRNAs are efficiently translated despite minimal transcriptional change. In potato, matched RNA-seq and ribosome-profiling (Ribo-seq) revealed stress-specific translational reprogramming under drought and heat, while tomato studies linked ribosome occupancy to fruit cracking and tissue-specific root development (Jian et al., 2023; Kajala et al., 2021; Zhong et al., 2024). However, direct evidence that phytochromes, cryptochromes or UVR8 control translational efficiency in *Solanaceae* remains to be developed. Links among spectral regime, TOR/SnRK1 activity, uORF-mediated control and selective translation should therefore be framed as testable mechanistic extensions supported by broader plant evidence on energy signaling and translational regulation (Baena-González and Hanson, 2017; Causier et al., 2022; Wu et al., 2024a).

Changes in ribosome loading frequently diverged from transcript abundance: transcripts strongly induced at the mRNA level were not necessarily translated, while others maintained efficient translation without upregulation. Moreover, genes involved in ribosome biogenesis, initiation factors, and tRNA metabolism exhibited stress-specific translation patterns, implying that the translation machinery itself is remodeled during acclimation (Jian et al., 2023). Combined RNA-seq and Ribo-seq analyses comparing cracking-resistant and susceptible tomato fruit revealed that many key genes in cell-wall metabolism, water transport, and stress signaling are regulated primarily at the level of translation rather than transcription (Zhong et al., 2024). Cell-type-resolved translatome profiling of tomato roots showed that developmental processes such as xylem differentiation depend on selective translation within specific cell populations, even when mRNA abundance is uniform (Kajala et al., 2021). Understanding translational behavior in reproductive and sink tissues—flowers, fruits, stolons, and tubers—is particularly important, since yield and quality reductions often originate from these organs rather than from vegetative stress.

Transcripts containing upstream open reading frames (uORFs) in their 5′ untranslated regions represent an additional layer of translational control. uORFs commonly attenuate translation of downstream main coding sequences by altering ribosome scanning, initiation, termination, reinitiation or stalling, although their effects can vary depending on uORF length, start-codon context, peptide sequence and transcript structure (van der Horst et al., 2020). Conserved peptide uORFs (CPuORFs) are particularly relevant because some encode short regulatory peptides that modulate ribosome behavior in response to metabolites or stress-related signals. For example, sucrose-responsive uORFs in the Arabidopsis S1-bZIP family mediate sucrose-induced repression of translation and thereby connect carbohydrate status with amino-acid and energy metabolism (Rahmani et al., 2009; van der Horst et al., 2020). Stress-responsive CPuORFs also regulate translation under heat and water-limitation conditions, supporting the view that uORFs can act as conditional translational gates during environmental stress (Causier et al., 2022).

uORFs are especially relevant to light-stress responses because light intensity and spectral quality alter photosynthetic carbon fixation, sugar availability, ATP status and amino-acid pools, all of which can influence translation initiation and reinitiation. In *Arabidopsis*, light enhances translation through a photoreceptor–COP1–auxin–TOR–RPS6 pathway, demonstrating that light signaling can directly affect translational capacity through energy- and growth-regulatory networks (Chen et al., 2018). In tomato, ribosome profiling has identified numerous translated uORFs and revealed conserved as well as *Solanaceae*-specific translational features, emphasizing the need to study translational regulation directly in crop genomes (Wu et al., 2019). Functional relevance is further supported by tomato studies showing that a peptide sequence-dependent regulatory uORF can repress translation of a tomato *ANAC096* homologue, and that CRISPR/Cas9 disruption of the sucrose-controlled uORF region of *SlbZIP1* increases soluble sugars and amino-acid contents in fruit (Noh et al., 2015; Nguyen et al., 2023). In potato, Ribo-seq detected thousands of uORFs and showed that uORF features significantly affect translational efficiency under drought and heat stress (Jian et al., 2023). This is particularly relevant for light-stress research because potato development is strongly photoperiod-sensitive, and controlled lighting affects both photosynthetic carbon supply and tuberization.

Thus, translational control provides a rapid and selective means for *Solanaceae* crops to adjust protein synthesis in response to dynamic light and stress conditions. The main evidence and remaining knowledge gaps are summarized in **Table 1**. Evidence from potato and tomato demonstrates that translation can be uncoupled from transcription, stress-specific, and highly tissue-focused (Jian et al., 2023; Kajala et al., 2021; Zhong et al., 2024). Establishing how photoreceptor signaling, TOR/SnRK1 dynamics, and uORF regulation converge on the translatome will position translation at the center of predictive light-stress biology.

**Table 1:** Evidence map linking light perception, RNA-regulatory layers and *Solanaceae* stress outcomes.

Core mechanism	Evidence highlighted in the review	Evidence status and main redouts
Photoreceptor signaling Red/far-red, blue and UV-B perception through PHYB1/PHYB2, CRY1a/CRY2 and UVR8; convergence through COP1/HY5/PIFs, hormone pathways, carbon balance and redox state.	In tomato, far-red light alters shoot:root allocation through PHYB1/PHYB2-dependent signaling and auxin transport (Ji et al., 2023), and far-red responses depend on background light intensity and the balance between photosynthetic input and phytochrome signaling state (Shomali et al., 2024). Tomato phytochromes also influence responses to nutrient deficiency (Soares et al., 2021), while CRY1a modulates osmotic and nutrient-stress responses under blue light (D’Amico-Damião et al., 2022). Tomato UVR8 regulates UV-B acclimation and fruit chloroplast traits (Liu et al., 2020; Li et al., 2018). UV-B/COP1/phyB/HFR1 thermomorphogenic crosstalk is mainly supported by model-plant evidence (Hwang et al., 2025).	Direct *Solanaceae* evidence for several photoreceptor-dependent physiological and stress responses; model-plant evidence for some COP1/PIF/HFR1 light–temperature crosstalk mechanisms. Controlled spectra, photoreceptor mutants, PSS/R:FR reporting, UV-B dose, blue-light fluence, physiological phenotyping, hormone/redox measurements and transcriptomics.
Alternative splicing Stress-responsive splice-site choice generates isoform switches, altered protein domains, subcellular targeting changes and nonsense-mediated decay.	Tomato shows stress-responsive AS under salt stress, phosphate starvation, combined water–nutrient stress and heat stress (Ruggiero et al., 2022; Satheesh et al., 2022; Gan et al., 2024; Rosenkranz et al., 2024). Tomato serine/arginine-rich proteins contribute to splicing homeostasis and thermotolerance (Rosenkranz et al., 2024). Pepper and potato provide emerging evidence for stress-associated splice-variant regulation (Kim et al., 2024; Glushkevich et al., 2022). Photoreceptor-dependent AS mechanisms, including phyB-associated splicing-factor regulation, CRY2–CIS1 control of thermosensory flowering and COP1–UAP56 regulation of AS, are mainly established in *Arabidopsis* (Kathare and Huq, 2021; Kathare et al., 2022; Zhao et al., 2022; Li et al., 2022).	Direct *Solanaceae* evidence for stress-responsive AS; model-plant evidence for photoreceptor-dependent AS mechanisms; *Solanaceae* hypothesis for direct light-receptor control of isoform switching under controlled light-stress regimes. Short- and long-read RNA-seq, splice-junction usage, ΔPSI, isoform-specific RT-PCR, NMD prediction, domain analysis and proteomic validation.
RNA stability, decay and condensates Transcript half-life, decapping, m^6^A-mediated fate decisions and partitioning into P-bodies or stress granules regulate transcript storage, degradation and recovery.	Tomato diurnal transcript dynamics are shaped by RNA metabolism (Zhang et al., 2023), and stress-associated m^6^A remodeling in tomato fruit links RNA stability with hormone signaling, cell-cycle regulation and carotenoid metabolism (Han et al., 2025). In Arabidopsis, DCP5-dependent condensates participate in osmotic-stress RNA regulation (Wang et al., 2024), ECT8 links m^6^A recognition with mRNA decay under stress (Cai et al., 2024), stress granules support recovery after heat stress, and phytochrome A mediates P-body disassembly under far-red light (Schwenk and Hiltbrunner, 2022). Light-dependent changes in mRNA decay and translational efficiency have also been demonstrated in model systems (Smith et al., 2024).	Partial direct *Solanaceae* evidence for RNA metabolism and m^6^A-associated transcript stability; model-plant evidence for DCP5, ECT8, PB/SG and phyA-linked condensate mechanisms; *Solanaceae* hypothesis for photoreceptor-dependent RNA-fate regulation. RNA half-life assays, transcriptional inhibition or metabolic labeling, m^6^A profiling, decapping/decay markers, PB/SG imaging, RNA capture from condensates and recovery-time profiling.
Small RNAs pathways miRNAs, siRNAs and phasiRNAs regulate target transcripts through cleavage and translational repression; feedback loops affect hormone, redox and stress-response networks.	Several *Solanaceae* miRNA modules are experimentally associated with stress responses: tomato miR162–DCL1 under low-night-temperature stress (Li et al., 2023), miR171d–GRAS24 in fruit chilling response (Xing et al., 2022), miR164a in salinity tolerance (Wan et al., 2023), miR172-related cleavage and translational repression under stress (Bansal et al., 2024), miR398 in thermotolerance (Li et al., 2025), and pepper miR397–laccase in anther heat response (Lei et al., 2026). Broader light/redox regulation of miRNA activity is supported by plant studies linking light status, redox state and miRNA function (Song et al., 2019; Gulyás et al., 2023), while UV-B–stress crosstalk through sRNA pathways is mainly model-derived (Zhou et al., 2024b).	Direct *Solanaceae* evidence for stress-associated miRNA modules; limited direct evidence for photoreceptor-dependent regulation of these modules; hypothetical *Solanaceae* relevance for spectrum-specific sRNA control under controlled environments. Small RNA-seq, degradome/PARE analysis, validated miRNA–mRNA pairs, AGO association, target mimicry, CRISPR or transgenic perturbation and ribosome-occupancy analysis for translational repression.
Translational control Ribosome loading, translational efficiency, uORFs and TOR/SnRK1 balance determine which mRNAs are converted into proteins during growth, stress and recovery.	Potato matched RNA-seq and Ribo-seq show stress-specific translational reprogramming under drought and heat (Jian et al., 2023). Tomato Ribo-seq/RNA-seq studies link translational regulation with fruit cracking and tissue-specific root development (Kajala et al., 2021; Zhong et al., 2024). TOR/SnRK1 signaling provides a broader plant framework connecting energy status, stress adaptation and translation (Baena-González and Hanson, 2017; Wu et al., 2024a), while uORFs act as conserved translational regulatory elements under stress (Causier et al., 2022; Urquidi Camacho et al., 2020). Tomato TOR inhibition is associated with enhanced cold tolerance and reduced chilling injury (Wu et al., 2024b; Yang et al., 2025).	Direct *Solanaceae* evidence for stress- and tissue-specific translational regulation; direct *Solanaceae* evidence for TOR-associated stress phenotypes; hypothetical/future direction for direct photoreceptor-dependent translational control under defined spectra. Ribo-seq, RNA-seq plus translational efficiency, polysome profiling, uORF annotation, ribosome-footprint quality metrics, TOR/SnRK1 activity assays and tissue-specific translatomics.
Machine-learning integration Multi-layer environmental, omics and phenotypic features are used to identify predictive modules and candidate mechanisms connecting light regimes with stress resilience.	General ML frameworks support multi-omics integration and interpretable prediction in biology (Libbrecht and Noble, 2015; Mahood et al., 2020; Panahi et al., 2025). In tomato, ML has identified drought-responsive biomarkers and reconstructed stress- or organ-specific regulatory networks (Chowdhury et al., 2023; Fernández et al., 2025). Methodological guidance emphasizes leakage-aware validation, interpretability, uncertainty reporting and external testing (Kapoor et al., 2024; Baião et al., 2025). Greenhouse decision-support and reinforcement-learning concepts provide future routes for adaptive environmental control (van Laatum et al., 2025; Padilla-Nates et al., 2025; Chen and You, 2026; Mallick et al., 2025).	Direct *Solanaceae* evidence for transcriptome-based ML and network inference; future research direction for integrated post-transcriptional, light-stress and multi-omics prediction. Feature engineering from ΔPSI, isoform ratios, validated miRNA–target pairs, m^6^A state, RNA half-life, translational efficiency, environmental time series, regularized regression, tree ensembles, network inference, SHAP and external validation.

## 6 Machine learning to integrate light perception with RNA-level regulation and stress phenotypes in vegetable *Solanaceae*

Elucidating how programmable light environments shape post-transcriptional RNA regulation and stress phenotypes in vegetable *Solanaceae* requires more than descriptive omics. It requires analytical frameworks that can account for the specific complexity of controlled-environment agriculture, where plants are exposed not simply to “light treatments” but to dynamic spectral histories. The machine-learning (ML) models for controlled-environment *Solanaceae* research should treat light as a multidimensional, time-dependent variable rather than as a simple categorical label such as “blue light”, “far-red” or “UV-B” (ANSI/ASABE S640, 2017/2022; Kusuma et al., 2020; Paradiso and Proietti, 2022). ML provides tools to integrate the heterogeneous datasets, identify predictive molecular features and define candidate regulatory modules linking spectral regimes with acclimation, recovery, yield stability and fruit quality (Libbrecht and Noble, 2015; Mahood et al., 2020; Panahi et al., 2025). ML and deep-learning approaches are increasingly used in CEA for crop monitoring, stress detection, growth prediction, microclimate management and decision support (Ojo and Zahid, 2022; Dsouza et al., 2023). In *Solanaceae* research, ML should therefore serve three main purposes: first, to identify RNA-level features that predict performance under defined spectral environments; second, to determine which features remain robust across genotypes, tissues, facilities and lighting systems; and third, to translate molecular signatures into testable hypotheses for spectrum design and crop management.

The main obstacle to predictive modeling in controlled-environment *Solanaceae* research is not only limited sample size, but also heterogeneity among datasets. Even when experiments use similar labels, such as “blue light”, “far-red supplementation” or “UV-B treatment”, the underlying biological exposure may differ substantially. If light treatments are encoded only as categorical variables, models may learn dataset-specific artifacts rather than general biological relationships (ANSI/ASABE S640, 2017/2022; Kusuma et al., 2020; Paradiso and Proietti, 2022). Environmental variables such as temperature, vapour-pressure deficit, CO_2_ concentration, irrigation and nutrient status should also be recorded as synchronized time series rather than as static background information. This is especially important because RNA-level responses are highly time dependent: an isoform switch, miRNA pulse or change in translational efficiency may reflect the preceding light history rather than the light condition at the exact sampling moment. Therefore, ML pipelines should explicitly include strategies for harmonization and domain-shift testing. Metadata should be structured using shared descriptors wherever possible, and environmental variables should be summarized in biologically meaningful time windows, such as 1 h, 6 h, 24 h or multi-day exposure before sampling. Standards for plant phenotyping metadata, including MIAPPE, provide useful principles for improving dataset reusability and cross-study comparison, even though controlled-environment light experiments require additional spectral descriptors beyond standard agronomic metadata (Papoutsoglou et al., 2020).

During model selection, the most interpretable features that correspond to known light-perception and RNA-regulatory mechanisms should be included. For alternative splicing, useful features include ΔPSI values, isoform ratios, intron-retention frequency, splice-junction usage and predicted AS–NMD coupling. For small RNAs regulation, features may include miRNA abundance, validated miRNA–target pairs, degradome-supported cleavage events and ratios between miRNAs and their target transcripts. For RNA stability and decay, relevant inputs include transcript half-life estimates, m^6^A state, abundance of decay-related transcripts and RNA-fate indicators. For translational regulation, features should include ribosome-loading indices, translational efficiency, uORF-associated regulation and stress-specific Ribo-seq signatures. Non-destructive phenotyping traits are widely used to monitor plant physiological status, stress responses and recovery dynamics, because RGB imaging, chlorophyll fluorescence imaging, thermal imaging and hyperspectral imaging can capture complementary aspects of growth, photosynthetic performance, water status and biochemical composition (Humplík et al., 2015; Mishra et al., 2020; Gill et al., 2022; Ye et al., 2023). Once validated, these non-destructive proxies could be used for monitoring plant status under different light regimes, while RNA-level markers remain mechanistic indicators used in calibration experiments. This approach would allow ML to connect post-transcriptional regulation with practical greenhouse monitoring without requiring routine destructive molecular sampling (Feng et al., 2020; Ojo and Zahid, 2022).

Unsupervised learning, including principal-component analysis, clustering, self-organizing maps and latent-factor models, is useful for detecting hidden structure in light × RNA datasets (Vahabi et al., 2022; Yu et al., 2024). It can reveal whether samples separate according to spectral regime, genotype, tissue, developmental stage, facility or batch. This is especially valuable for identifying unwanted confounding. For example, if samples cluster primarily by greenhouse facility rather than by light treatment, a supervised model may learn facility-specific conditions rather than biological light responses. Semi-supervised or weakly supervised approaches may be valuable when labels are incomplete, as is common for isoform function, miRNA targets or recovery phenotypes in non-model crops (Yan et al., 2022; Tran et al., 2022).

Single-omics models can identify correlations, but controlled-environment light responses are produced by cascades of perception, metabolism and RNA regulation. General multi-omics literature emphasizes that integration strategy, missing-value handling, normalization, scaling and batch correction strongly influence model outcomes (Ritchie et al., 2015; Huang et al., 2017; Mahood et al., 2020). In controlled-environment *Solanaceae* studies, intermediate integration is often most biologically interpretable because it preserves the structure of each regulatory layer while allowing cross-layer relationships to be tested. Mechanistically informative models should prioritize regulatory modules rather than isolated markers, because multi-omics integration is most useful when it links coordinated molecular features to biological processes rather than selecting single high-ranking variables (Ritchie et al., 2015; Huang et al., 2017; Subramanian et al., 2020; Mahood et al., 2020). Examples include an isoform-ratio module predicting recovery under far-red × heat conditions, a miRNA–target module associated with blue-light-conditioned osmotic acclimation, an m^6^A-associated decay module linked with fruit-quality maintenance, or a translational-efficiency module predicting heat recovery in potato.

Graph-based models may be especially useful for representing relationships among photoreceptors, environmental variables, RNA-regulatory features and phenotypes. Graph neural networks and related approaches can incorporate prior biological knowledge, such as known miRNA–target pairs, transcription-factor networks, protein–protein interactions or metabolic pathways (Zitnik et al., 2018; Hegde et al., 2025). However, these models should be used cautiously in small plant datasets because highly flexible architectures can infer spurious relationships. The most useful graph models for *Solanaceae* light biology will be those that combine data-driven learning with prior knowledge and experimental validation.

**Figure 4:**
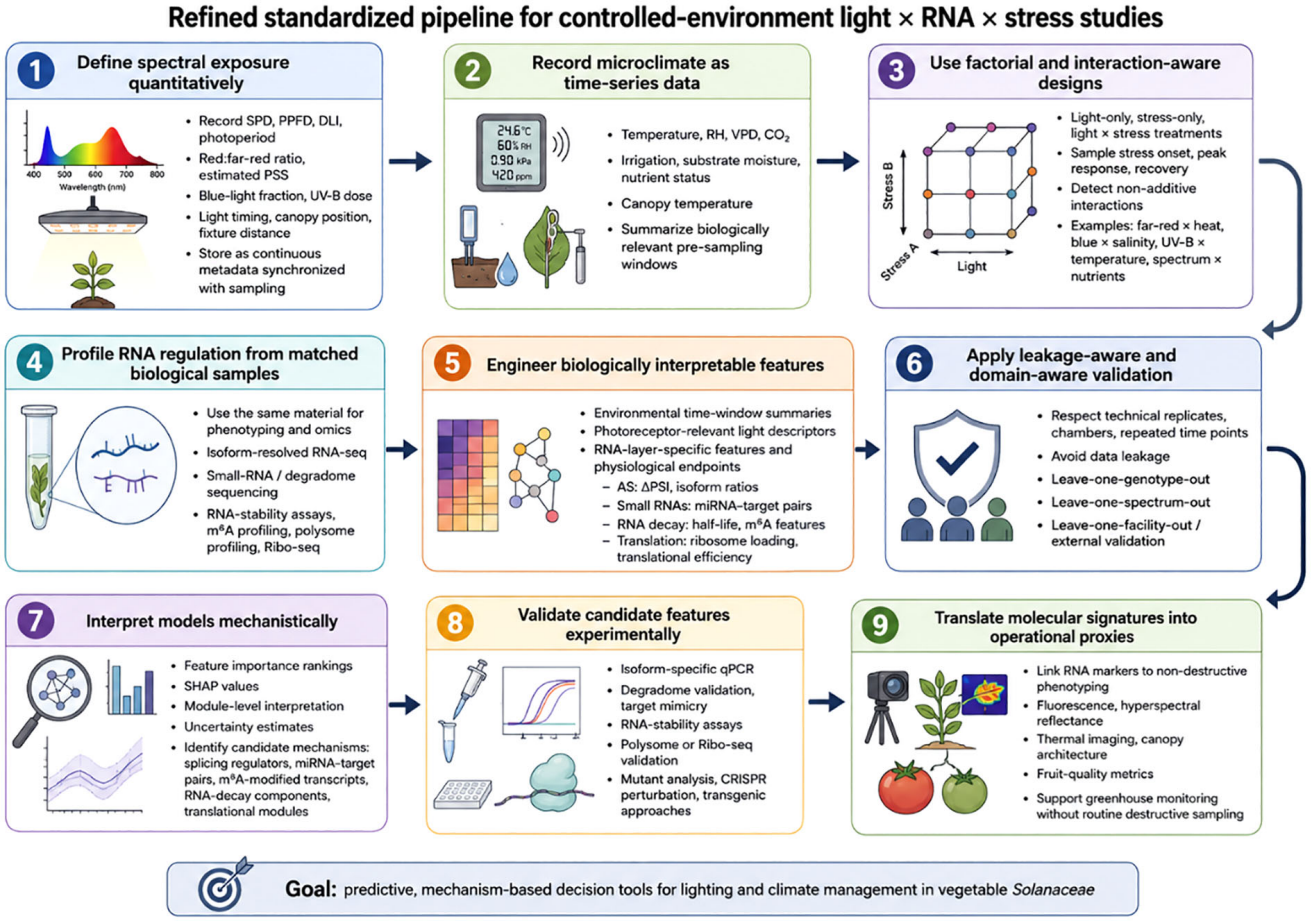
Standardized pipeline for controlled-environment light × RNA × stress studies in vegetable *Solanaceae*. The schematic presents a nine-stage workflow linking quantitative light characterization, microclimate monitoring, factorial light-stress designs, matched RNA profiling, interpretable feature engineering, leakage-aware validation, mechanistic model interpretation, experimental validation and non-destructive phenotyping proxies. The pipeline emphasizes that spectral exposure and microclimate should be recorded as synchronized metadata, RNA-level features should be connected to physiological traits, and ML-derived markers should be experimentally validated before being used for lighting and climate-management decisions.

Reinforcement-learning and model-predictive-control frameworks also provide future routes for dynamic greenhouse optimization, especially where environmental decisions must be made sequentially over time (van Laatum et al., 2025; Padilla-Nates et al., 2025; Chen and You, 2026; Mallick et al., 2025). The **Table 2** presents the summary of machine-learning approaches relevant to plant stress biology and Solanaceae light × RNA studies and **Figure 4** shows a standardized pipeline for controlled light – RNA – stress studies. Overall, ML should be presented not as an autonomous solution, but as a framework for organizing heterogeneous light, RNA and phenotype data into testable biological models.

**Table 2:** Summary of machine-learning approaches relevant to plant stress biology and *Solanaceae* light × RNA studies.

ML approach	Typical algorithms	Main input data	Main use in plant stress biology	Interpretability/feature-selection strategy	Main limitations	Representative references
Supervised classification	Logistic regression, support-vector machines, random forests, gradient boosting, neural networks	Transcriptomes, metabolomes, small RNAs profiles, phenotyping images, hyperspectral data, environmental metadata	Classification of control versus stressed plants; discrimination among drought, heat, salinity, chilling or nutrient stress; identification of diagnostic stress biomarkers	LASSO/elastic net coefficients, tree-based feature importance, recursive feature elimination, SHAP, confusion matrices and external validation	Requires reliable labels; sensitive to class imbalance, batch effects and data leakage; models may learn facility-specific artefacts rather than general stress biology	Libbrecht and Noble (2015); Mahood et al. (2020); Chowdhury et al. (2023); Kapoor et al. (2024); Paul et al. (2025)
Supervised regression	Ridge regression, LASSO, elastic net, random-forest regression, gradient-boosting regression, partial least squares regression	Omics features, environmental time series, biomass, yield, fruit quality, recovery indices	Prediction of quantitative stress severity, growth reduction, recovery time, yield stability or fruit-quality traits	Regression coefficients, permutation importance, SHAP, residual diagnostics, calibration plots	Requires continuous phenotypes measured consistently across experiments; performance can decline under genotype or facility domain shift	Libbrecht and Noble (2015); Mahood et al. (2020); Chowdhury et al. (2023); Kapoor et al. (2024)
Unsupervised clustering and dimensionality reduction	PCA, hierarchical clustering, k-means, self-organizing maps, t-SNE, UMAP, latent-factor models	Transcriptomics, metabolomics, proteomics, phenomics, environmental sensor data	Discovery of stress-response groups, genotype clusters, treatment gradients, outliers, batch effects and co-regulated molecular modules without predefined labels	Cluster stability, silhouette scores, loading plots, marker-feature enrichment, biological annotation of clusters	Does not directly prove stress causality; clusters may reflect batch, tissue or developmental differences rather than stress response	Libbrecht and Noble (2015); Baião et al. (2025); Panahi et al. (2025)
Feature selection and biomarker discovery	Variance filtering, correlation filtering, mutual information, LASSO, elastic net, Boruta, recursive feature elimination, random-forest importance, SHAP ranking	High-dimensional RNA-seq, isoform ratios, miRNAs, metabolites, Ribo-seq, phenotypic traits	Reduction of large omics datasets to compact biomarker sets; prioritization of genes, isoforms, miRNAs or metabolites for validation	Stability selection across resampling, external validation, SHAP, biological enrichment analysis, independent RT-qPCR or functional validation	Biomarkers can be unstable when sample size is small; correlated features can distort rankings; selected markers may not be causal	Libbrecht and Noble (2015); Chowdhury et al. (2023); Kapoor et al. (2024)
Gene-regulatory-network inference	GENIE3, random-forest network inference, co-expression networks, Bayesian networks, graphical models	Gene expression, transcription-factor lists, miRNA–target pairs, isoform data, environmental variables	Identification of regulatory hubs, stress-response modules and candidate upstream regulators linking light perception with RNA-level regulation	Edge confidence, hub ranking, module enrichment, comparison with known pathways, perturbation validation	Network edges often indicate association, not causality; sensitive to sample size, time-point density and hidden confounders	Huynh-Thu et al. (2010); Mahood et al. (2020); Fernández et al. (2025)
Isoform-aware and sequence-informed ML	Positive–unlabeled learning, random forests, gradient boosting, sequence-feature models, deep learning	Long-read transcriptomes, short-read splice junctions, ΔPSI, UTR length, uORFs, miRNA sites, RNA structure	Prioritization of functionally relevant splice isoforms, prediction of translational potential, identification of RNA features associated with stress responses	Isoform-level feature importance, domain-gain/loss prediction, NMD prediction, isoform-specific RT-PCR, proteomic confirmation	Requires high-quality annotation; short-read data can misassign isoforms; functional validation remains essential	Qi et al. (2022); Xu et al. (2024); Causier et al. (2022); Jian et al. (2023)
Multi-omics integration	Early feature concatenation, multi-block PLS, latent-factor models, autoencoders, graph-based integration, ensemble models	Matched transcriptomics, small RNAs/degradome, m^6^A, metabolomics, Ribo-seq, proteomics, phenotyping and environmental metadata	Identification of cross-layer modules connecting environmental exposure, RNA regulation and stress phenotype; separation of transcriptional, post-transcriptional and metabolic effects	Module-level interpretation, joint embeddings, pathway enrichment, layer-specific feature importance, external validation	Missing data, incompatible normalization, batch effects and unequal sample sizes across omics layers can reduce robustness	Libbrecht and Noble (2015); Baião et al. (2025); Panahi et al. (2025)
Deep learning for phenotyping and stress detection	Convolutional neural networks, recurrent neural networks, transformers, autoencoders, multimodal neural networks	RGB images, thermal images, fluorescence, hyperspectral data, time-series sensor data, sometimes omics data	Non-destructive detection of drought, heat, salinity, disease or nutrient stress; extraction of physiological proxies for molecular stress states	Saliency maps, Grad-CAM, attention weights, feature embeddings, external test sets	High data demand; limited interpretability; prone to overfitting and poor transfer across cameras, cultivars, facilities and seasons	Paul et al. (2025); Kapoor et al. (2024); Baião et al. (2025)
Reinforcement learning and decision-support models	Q-learning, actor–critic methods, model-predictive control with RL, policy-gradient methods	Greenhouse climate data, irrigation data, lighting schedules, crop-growth models, phenotypic feedback	Optimization of irrigation, climate and potentially lighting strategies for stress mitigation and resource efficiency	Reward decomposition, constraint monitoring, policy evaluation under unseen weather or facility conditions	Not yet commonly linked to molecular data; risk of optimizing short-term growth at the expense of long-term resilience; requires realistic simulation or safe deployment protocols	van Laatum et al. (2025); Padilla-Nates et al. (2025); Mallick et al. (2025)

## 7 Methodological constraints and future directions for predictive light stress biology in vegetable *Solanaceae*

The key methodological constraints and the future directions are summarized in the **Table 3**. A first priority for future research is the complete reporting of environmental exposure histories. Many studies still describe treatments using broad labels such as “blue light”, “far-red supplementation”, “UV-B treatment”, “heat stress” or “salt stress”, without providing the quantitative information required to reconstruct biological exposure. Critical variables include spectral power distribution, photosynthetic photon flux density (PPFD), absolute blue photon flux, red:far-red ratio, phytochrome photostationary state (PSS), UV-B dose rate and cumulative dose, photoperiod, canopy-level irradiance, leaf temperature, VPD, irrigation cycles, nutrient composition and developmental timing. Such omissions limit reproducibility, meta-analysis and mechanistic interpretation. Far-red radiation, for example, can act either as an additional photosynthetic input or as a phytochrome signal depending on background irradiance and spectral balance (Shomali et al., 2024), while its developmental timing can determine whether tomato fruit growth and ripening are promoted or delayed (Mastoraki et al., 2025). Similarly, blue-light effects are fluence-rate dependent, particularly for CRY1a-mediated responses (D’Amico-Damião et al., 2022), and UV-B outcomes depend strongly on dose, timing and acclimation history (Liu et al., 2020; Hwang et al., 2025). Controlled environments therefore do not remove variability; they create a need to measure it precisely. MIAPPE 1.1 provides a useful framework for harmonized phenotyping metadata, and broader analyses of plant data reuse emphasize metadata completeness as a prerequisite for reproducibility and interoperability (Papoutsoglou et al., 2020; Danilevicz et al., 2021). Applying MIAPPE-aligned templates to *Solanaceae* light-stress studies would make it easier to distinguish true spectral effects from hidden environmental covariates.

**Table 3:** Recommended experimental and modeling framework for future light x RNA x stress studies in vegetable *Solanaceae*.

Stage	Minimum recommendation	Data generated	Analysis/model	Validation endpoint
1. Define exposure history	Record spectral power distribution, PPFD, R:FR ratio/PSS, blue-light fraction, UV-B dose, photoperiod, canopy position, temperature, VPD, irrigation, nutrients and developmental stage as time series.	Continuous environmental metadata synchronized with sampling time.	Metadata harmonization; MIAPPE-aligned descriptors; time-window summaries.	Reproducible treatment reconstruction and cross-study comparability.
2. Use factorial treatment structure	Include control, light-only, stress-only and combined light-stress treatments; sample stress onset, peak stress and recovery.	Interaction-aware experimental matrix with temporal resolution.	ANOVA or mixed models for interactions; time-series modeling.	Separation of direct spectral effects from stress effects and non-additive interactions.
3. Profile RNA regulation in matched samples	Measure at least one RNA layer from the same biological material used for phenotyping: AS, RNA half-life/m^6^A, small RNA/degradome or Ribo-seq.	Isoform ratios, transcript-fate metrics, miRNA-target evidence or translational efficiency values.	Layer-specific pipelines plus integrated feature matrices.	Mechanistic link between RNA layer and phenotype.
4. Prioritize causal tissues	Move beyond leaves to roots, anthers, flowers, fruit pericarp, stolons and tubers where yield and quality losses originate.	Tissue-specific molecular and physiological datasets.	Cell-/tissue-aware differential analysis; network modules.	Crop-relevant endpoints such as root water control, pollen fertility, fruit cracking, ripening or tuber development.
5. Build interpretable predictive models	Use biologically constrained features such as ΔPSI, validated miRNA-target pairs, m^6^A-modified transcripts, half-life estimates and translational-efficiency modules.	Compact feature sets linked to environmental variables and phenotypes.	Regularized regression, random forest, gradient boosting, network inference and SHAP-based explanation.	Stable candidate modules that predict resilience or recovery across replicates.
6. Test generalization	Validate across cultivar, facility, light hardware, season and independent experimental batches.	External test sets and uncertainty estimates.	Leakage-aware splitting; domain-shift assessment; calibration analysis.	Transferable models rather than facility-specific classifiers.
7. Experimentally validate top modules	Confirm top features using isoform-specific RT-PCR, degradome validation, target mimicry, CRISPR/transgenics, photoreceptor mutants or targeted Ribo-seq.	Causal evidence for regulatory modules.	Perturbation-response modeling and mechanistic network refinement.	Photoreceptor -> RNA-regulatory layer -> phenotype chain.
8. Translate to management	Use validated RNA modules to identify physiological proxies such as fluorescence, thermal imaging, hyperspectral markers or metabolite signatures.	Non-destructive proxy data linked to molecular ground truth.	Decision-support, model-predictive control or reinforcement-learning prototypes.	Actionable spectrum scheduling and stress mitigation in CEA.

Experimental design must also move beyond single-factor comparisons. Many studies impose light modification and stress simultaneously but omit spectrum-only and stress-only controls, making it difficult to distinguish direct spectral effects, direct stress effects and non-additive interactions. This distinction is essential because photoreceptors can actively shape stress physiology rather than simply modify growth conditions. Robust experiments should therefore include untreated controls, light-only treatments, stress-only treatments and combined light-stress treatments, with sampling during stress onset, peak response and recovery. Time-resolved sampling is especially important because RNA decay, processing-body and stress-granule dynamics, and translational regulation can change rapidly after perturbation (Wang et al., 2024; Smith et al., 2024). Single terminal time points may therefore confuse transient regulatory events with stable acclimation.

A further challenge is that most molecular studies still emphasize accessible leaf tissue, whereas yield losses in vegetable *Solanaceae* often originate in roots, flowers, fruits, stolons and tubers. These organs are where stress tolerance, reproductive success, storage-organ formation and fruit quality converge. Tomato drought tolerance, for example, depends on a suberized exodermis that modifies whole-plant water management (Cantó-Pastor et al., 2024), while pepper reproductive heat tolerance involves small RNAs-mediated regulation of lignin biosynthesis in anthers (Lei et al., 2026). In tomato fruit, translational regulation contributes to cell-wall remodeling, cracking resistance and tissue-specific development (Kajala et al., 2021; Zhong et al., 2024), and integrated transcriptomic–metabolomic analyses continue to reveal regulators of ripening and quality traits (Dong et al., 2025). Future light-stress studies should therefore prioritize causal tissues and match RNA-level measurements with physiological and agronomic endpoints.

At the transcriptome level, short-read RNA-seq remains valuable but insufficient for resolving full isoform complexity. Splice-junction detection varies across pipelines, and reads from repetitive regions, overlapping transcripts or highly similar gene families can be difficult to assign unambiguously (Tu et al., 2022; Jiang et al., 2023b). Tomato studies have identified extensive alternative-splicing responses to salinity, phosphate limitation, heat and combined stresses (Ruggiero et al., 2022; Satheesh et al., 2022; Gan et al., 2024; Rosenkranz et al., 2024), but functional validation remains limited. Long-read sequencing can resolve full-length isoforms and reduce junction ambiguity, although it may also reveal low-abundance or incompletely processed transcripts of uncertain relevance. Tools such as iFLAS can help prioritize functionally plausible isoforms, but key claims should be supported by isoform-specific RT-PCR, nonsense-mediated decay prediction, domain analysis, proteomic evidence or perturbation experiments (Xu et al., 2024b). Isoform-resolved studies should therefore be embedded within factorial designs and synchronized with phenotyping, rather than treated as independent descriptive surveys.

RNA stability, condensate dynamics, small RNAs activity and translational control require similar validation standards. Steady-state transcript abundance reflects both synthesis and decay, yet direct RNA-stability measurements remain uncommon in crops. Processing bodies and stress granules add further methodological complexity because their formation is sensitive to sample preparation, osmotic status, energy balance and imaging conditions. Model-species studies have clarified plant processing-body and stress-granule composition (Solis-Miranda et al., 2023; Kearly et al., 2024), but comparable data in vegetable *Solanaceae* remain scarce. Small RNAs studies also often rely on computational target prediction, which can generate false positives because miRNA–target pairing, AGO loading and cleavage efficiency vary by tissue and stress context. Stronger evidence requires degradome sequencing, target mimicry, ribosomal association assays, transgenic perturbation or genome editing (Ueno et al., 2022; Puchta-Jasińska et al., 2025). Updated degradome protocols that recover informative cleavage signatures from partially degraded RNA may improve feasibility in complex crop tissues such as fruit pericarp, pollen and stressed leaves (Dong et al., 2025).

Translational regulation is another critical but technically demanding layer. Ribo-seq provides direct information on ribosome occupancy and translational efficiency, making it uniquely useful for separating transcriptional regulation from protein-synthesis potential. However, it remains expensive, labor-intensive and sensitive to variation in tissue stabilization, nuclease digestion, ribosome-protected fragment isolation, rRNA depletion, footprint-size selection and normalization (Jian et al., 2023; Ting et al., 2024; Zhong et al., 2024). These challenges increase in crop tissues rich in secondary metabolites or polysaccharides and in large factorial studies involving multiple genotypes, tissues and recovery stages. Improved and low-input plant Ribo-seq protocols increase feasibility for delicate tissues, but broad deployment across *Solanaceae* panels remains difficult (Ting et al., 2024; Tomuro and Iwasaki, 2025). A practical strategy is to apply Ribo-seq selectively after lower-cost physiological phenotyping, RNA-seq or polysome-based screening has identified the most informative genotypes, organs and time points.

Because no single omics layer can explain light-conditioned stress responses, future studies should connect regulatory layers into sequential pathways rather than parallel datasets. Instead of describing light, stress and expression independently, experiments should test causal chains such as light exposure → photoreceptor state → splicing-factor activity → isoform switching → recovery; blue-light fluence → redox and ABA status → miRNA expression → translational repression → osmotic acclimation; or far-red × heat interaction → TOR/SnRK1 modulation → translational efficiency of protective transcripts → recovery growth. Building such pathways requires synchronized sampling of multiple data layers from the same biological material, including RNA-seq, long-read transcriptomics, small RNAs sequencing, degradome analysis, m^6^A profiling, RNA-stability assays, Ribo-seq, metabolomics, proteomics and phenotyping. Integrated transcriptome–metabolome analyses already show how matched datasets can improve mechanistic inference in tomato fruit ripening and stress biology (Dong et al., 2025; Yu et al., 2025).

Adaptive greenhouse management is the long-term application of this work, but molecular prediction must be translated carefully. Reinforcement learning, digital twins and hybrid model-predictive control are increasingly being explored for greenhouse climate and irrigation management (van Laatum et al., 2025; Padilla-Nates et al., 2025; Chen and You, 2026; Mallick et al., 2025). In principle, biologically informed controllers could adjust spectrum, intensity, photoperiod or irrigation according to predicted molecular or physiological state. In practice, destructive sequencing is too costly and delayed for real-time control. The near-term goal should therefore be to use RNA-level datasets as molecular ground truth for identifying non-destructive proxies, including canopy temperature, chlorophyll fluorescence, hyperspectral signatures, architectural traits, fruit color and metabolite markers. These proxies could then support adaptive lighting and climate strategies while validated RNA-level signatures remain benchmarks for calibration and interpretation.

## 8 Conclusion

The evidence reviewed here indicates that RNA-level regulation is essential for explaining why transcript abundance alone often fails to predict stress tolerance, recovery capacity or yield stability. Alternative splicing modifies transcript and protein diversity; RNA stability and decay regulate transcript persistence and recovery dynamics; small RNAs control target transcripts through cleavage or translational repression; and translational regulation determines which mRNAs are converted into proteins under stress. Together, these mechanisms provide the flexibility required for *Solanaceae* crops to respond rapidly to changing combinations of light, temperature, water status, nutrients and developmental cues.

Current evidence is strongest in tomato, where stress-responsive alternative splicing, RNA metabolism, miRNA regulation, m^6^A-associated transcript stability and translational control have all been documented. Pepper and potato studies also reveal important RNA-level responses, particularly in reproductive heat stress and drought- or heat-induced translational reprogramming. However, direct evidence linking defined photoreceptor pathways with post-transcriptional regulation in Solanaceae remains limited, and this should become a priority for future work.

Progress will depend on experiments that combine precise environmental metadata, factorial light-stress designs, time-resolved sampling and crop-relevant tissues such as roots, flowers, fruits, anthers, stolons and tubers. Integrating these datasets with interpretable machine-learning approaches can help identify regulatory modules—such as isoform switches, miRNA–target pairs, RNA-stability signatures and translational-efficiency markers—that connect light regimes with stress resilience and crop performance. Such integration will move controlled-environment Solanaceae research from descriptive omics toward predictive, mechanism-based crop management.

## Glossary

ABA: Abscisic acid
AGO: ARGONAUTE protein
AS: Alternative splicing
CEA: Controlled-environment agriculture
COP1: CONSTITUTIVELY PHOTOMORPHOGENIC 1
CPuORF: Conserved peptide upstream open reading frame
CRY: Cryptochrome
DCL: DICER-LIKE protein
FR: Far-red light
GA: Gibberellin
GRN: Gene-regulatory network
HFR1: LONG HYPOCOTYL IN FAR-RED 1
HY5: ELONGATED HYPOCOTYL 5
ML: Machine learning
m⁶A: N⁶-methyladenosine
miRNA: microRNA
NMD: Nonsense-mediated decay
PARE: Parallel analysis of RNA ends
PB/P-body: Processing body
PHY: Phytochrome
PIF: PHYTOCHROME-INTERACTING FACTOR
PPFD: Photosynthetic photon flux density
PSS: Phytochrome photostationary state
R:FR: Red:far-red ratio
RBP: RNA-binding protein
Ribo-seq: Ribosome profiling/ribosome-footprint sequencing
RISC: RNA-induced silencing complex
RL: Reinforcement learning
RNA-seq: RNA sequencing
ROS: Reactive oxygen species
RT-PCR: Reverse-transcription polymerase chain reaction
SG: Stress granule
SHAP: SHapley Additive exPlanations
siRNA: Small interfering RNA
SnRK1: SNF1-RELATED KINASE 1
sRNA: Small RNA
TOR: TARGET OF RAPAMYCIN
uORF: Upstream open reading frame
UV-B: Ultraviolet-B radiation
UVR8: UV RESISTANCE LOCUS 8
VPD: Vapour-pressure deficit
ΔPSI: Change in percent spliced in.
