# Supplementary material for: Post-transcriptional regulation of light-stress responses and predictive modeling in vegetable Solanaceae Crops
Source: Front Plant Sci. 2026 Jul 7;17:1880044. doi: 10.3389/fpls.2026.1880044 (PMC13384934; doi:10.3389/fpls.2026.1880044)
Supplement: Supplementary file 1 [file Table1.docx]

| Table S1. Photoreceptors, associated RNA-regulatory pathways, abiotic stresses and physiological outcomes in Solanaceae crops. | | | | | |
| --- | --- | --- | --- | --- | --- |
| Photoreceptor / light signal | **Direct Solanaceae evidence** | **Associated RNA-regulatory pathway** | **Abiotic-stress context** | **Major physiological outcome** | **Evidence status** |
| PHYB1 / PHYB2; red/far-red balance; R:FR; PSS | Tomato PHYB1/PHYB2 mediate far-red-dependent shoot:root allocation through auxin transport. Tomato phytochromes also influence growth and oxidative status under nutrient limitation (Ji et al., 2023; Soares et al., 2021). | Alternative splicing is a plausible downstream layer because Arabidopsis phyB interacts with splicing-factor complexes that regulate photomorphogenic AS (Kathare and Huq, 2021; Kathare et al., 2022). Far-red-linked P-body disassembly has also been shown through phyA in Arabidopsis (Schwenk and Hiltbrunner, 2022). | Nutrient limitation, altered R:FR, far-red enrichment, heat or shade-like conditions. | Biomass partitioning, elongation, shoot:root allocation, nutrient-stress adjustment, fruit-development responses. | **Direct Solanaceae evidence** for physiological effects; **model-plant evidence** for photoreceptor-linked AS and P-body mechanisms. |
| CRY1a / CRY2; blue light; blue-light fluence | Tomato CRY1a modulates osmotic-stress and nutrient-deficiency responses under blue light, with fluence-rate dependence (D’Amico-Damião et al., 2022). | Small-RNA pathways and translational control are plausible downstream interfaces because blue light affects photosynthesis, redox state, stomatal behavior and hormone signaling. CRY2-dependent AS through CIS1 and *FLM* is established in Arabidopsis (Zhao et al., 2022). | Osmotic stress, nutrient deficiency, high blue-light fraction, photoperiod or fluence-rate changes. | Stomatal regulation, growth adjustment, nutrient accumulation, osmotic acclimation. | **Direct Solanaceae evidence** for CRY1a-dependent stress physiology; **model-plant evidence** for CRY2-linked AS; **hypothesis** for direct Solanaceae CRY-to-RNA regulation. |
| UVR8; UV-B radiation | Tomato SlUVR8 regulates UV-B photomorphogenesis, UV-B tolerance and fruit chloroplast differentiation through SlGLK2-related pathways. SlRUP and UVR8 nuclear-localization dynamics regulate signal duration (Li et al., 2018; Liu et al., 2020; Zhang et al., 2021; Fang et al., 2022). | RNA stability, small-RNA pathways and condensate-related processes are candidate downstream layers. UV-B–small-RNA crosstalk and UVR8/COP1/phyB/HFR1 temperature interactions are mainly supported by model-plant evidence (Zhou et al., 2024; Hwang et al., 2025). | UV-B exposure, high irradiance, heat, oxidative stress, fruit-development conditions. | UV-B acclimation, photoprotection, thermomorphogenic adjustment, chloroplast differentiation, fruit-quality traits. | **Direct Solanaceae evidence** for UVR8 physiological roles; **model-plant evidence** for UV-B × temperature and UV-B × sRNA crosstalk. |
| COP1 / HY5 / PIF signaling hub; convergence of red, blue and UV-B pathways | COP1/HY5/PIF components are conserved light-signaling regulators, but direct Solanaceae evidence linking this hub to RNA-regulatory layers remains limited. | COP1 interacts with RNA-processing regulation in Arabidopsis, including COP1–UAP56-associated AS control. COP1 also participates in UVR8/phyB/HFR1 light–temperature crosstalk (Li et al., 2022; Hwang et al., 2025). | Light × temperature interactions, UV-B × heat, shade-like spectra, combined stress. | Photomorphogenesis, thermomorphogenesis, stress acclimation, growth-defense balance. | **Model-plant evidence** for direct RNA-regulatory links; **future Solanaceae validation needed**. |
| Photoreceptor state as an integrated signal; combined spectra, photoperiod and intensity | In tomato, spectral effects depend on background light intensity, photoperiod, genotype and developmental stage. Far-red, blue and UV-B pathways influence stress physiology through hormone, carbon and redox networks (Shomali et al., 2024; D’Amico-Damião et al., 2022; Liu et al., 2020). | Multiple RNA layers may respond indirectly: AS, RNA stability/decay, small-RNA regulation and translational control. Direct Solanaceae evidence is strongest for stress-responsive RNA regulation, not yet for photoreceptor-dependent RNA regulation (Gan et al., 2024; Rosenkranz et al., 2024; Li et al., 2023; Wan et al., 2023; Jian et al., 2023; Zhong et al., 2024). | Heat, salinity, drought, chilling, nutrient limitation, combined light × stress regimes. | Acclimation, recovery capacity, yield stability, fruit quality, genotype-specific stress resilience. | **Direct Solanaceae evidence** for RNA regulation under stress; **hypothesis** for direct photoreceptor-to-RNA control under defined spectra. |
